# Eomes transcription factor is required for the development and differentiation of invariant NKT cells

**DOI:** 10.1038/s42003-019-0389-3

**Published:** 2019-04-29

**Authors:** Kanako Shimizu, Yusuke Sato, Masami Kawamura, Hiroshi Nakazato, Takashi Watanabe, Osamu Ohara, Shin-ichiro Fujii

**Affiliations:** 1 Laboratory for Immunotherapy, RIKEN Center for Integrative Medical Sciences (IMS), Yokohama, Kanagawa 230-0045 Japan; 2 Laboratory for Integrative Genomics, RIKEN Center for Integrative Medical Sciences (IMS), Yokohama, Kanagawa 230-0045 Japan

**Keywords:** Innate immunity, Cytokines

## Abstract

Eomes regulates the differentiation of CD8^+^ T cells into effector and memory phases. However, its role in invariant (i)NKT cells remains unknown. Here, we show the impact of Eomes on iNKT cells in the thymus and peripheral tissue using conditional knockout (Eomes-cKO) mice. In the thymus, CD1d-tetramer^+^CD24^+^CD44^−^NK1.1^−^CD69^+^stage 0 iNKT cells express higher levels of Eomes than the other iNKT stages. We also found that Eomes regulates NKT1 cell differentiation predominantly. Interestingly, the expression of Eomes in the steady state is low, but can be upregulated after TCR stimulation. We also showed epigenetic changes in the *Eomes* locus after activation. In addition, vaccination of C57BL/6, but not Eomes-cKO mice with iNKT ligand-loaded dendritic cells generated KLRG1^+^iNKT cells in lung, characterized as effector memory phenotype by transcriptome profiling. Thus, Eomes regulates not only the differentiation of NKT1 cells in the thymus, but also their differentiation into memory-like KLRG1^+^iNKT cells in the periphery.

## Abstract (web summary)

Kanako Shimizu, Yusuke Sato et al. examine the impact of the Eomes transcription factor on invariant NKT (iNKT) cell differentiation using conditional knockout mice. They show that Eomes is needed for the development of iNKT1 cells in the thymus, and for their activation and differentiation in peripheral tissues.

## Introduction

Invariant (i)NKT cells express an invariant T cell antigen receptor (TCR) α-chain and recognize a complex consisting of the antigen-presenting MHC-like molecule CD1d and a glycolipid^1,2^. Recent discoveries of key transcription factors and gene-expression programs have contributed to a deeper understanding of the iNKT cell lineage^3^. In the commitment to the NKT cell lineage, iNKT precursors are positively selected by CD4^+^CD8^+^ thymocytes which express CD1d-glycolipod complexes^4^. After the selection, the TCR signaling initially activates the calcineurin pathway and stimulates the transcription factors, NFAT and Egr2^5^. Then, Egr2 activates PLZF as the iNKT cell master regulator and also induces the common β subunit of the IL-2/IL-15 receptor (CD122). This leads to the subsequent steps of iNKT cell development, composed of cytokine expression and proliferation in response to the IL-15-CD122 axis^6^. Functional subsets of iNKT cells, NKT1, NKT2, and NKT17, have been reported to arise during the thymus to peripheral differentiation stages. The key transcription factors for NKT1, NKT2 and NKT17 development have been identified as T-bet, Gata3 and Rorγt, respectively^3^. However, these iNKT cell subsets can also undergo further differentiation in the periphery.

We previously reported that a population of Klrg1^+^ iNKT cells elicited after administration of α-GalCer-pulsed DCs (DC/Gal) are long-lived. These cells predominantly express the NKT1-like cytokine IFN-γ, cytotoxic molecules (FasL and granzyme A/B) and in particular express *Eomes*. Moreover when transferred to iNKT cell-deficient mice, they can respond to the same antigen quickly and robustly as a secondary memory type response^7^. However, the molecular mechanism by which the Klrg1^+^ iNKT population is generated after administration of DC/Gal remains unknown.

Eomesodermin (Eomes) is a T-box transcription factor with high homology to T-bet and is expressed by activated CD8^+^ T cells as well as in resting and activated NK cells^8^. Eomes is not expressed by naïve T cells, but plays an important role in the formation of conventional memory CD8^+^ T cells^9^. In particular, Eomes favours the development of central memory cells, characterized by longer survival and the important potential for homeostatic proliferation^10^. In addition, Eomes functions as a master regulator of cell-mediated immunity capable of controlling the expression of genes encoding essential effector molecules, such as IFN-γ, granzyme B, perforin, CXCR3, and CXCR4^9–13^. In addition, Eomes has recently been shown to regulate a specific population of CD8^+^ T cells in the thymus^14^. This population, which is called IL-4-induced innate memory CD8^+^ T cells shows striking upregulation of Eomes but not the related T-box factor, T-bet^14^. These findings suggest additional independent roles for Eomes in the regulation of particular T cell populations in both the thymus and peripheral tissues. However the function of Eomes by itself in iNKT cells remains to be demonstrated.

To this end, through studies of T cell-specific Eomes conditional knockout (Eomes-cKO) mice, we found that Eomes selectively played a role in NKT1 cell development in the thymus. This impaired development of NKT1 cells in Eomes cKO mice are due to a cell-autonomous effect, as demonstrated by bone marrow (BM) chimera study. Furthermore, when activated, the remaining iNKT cells in the peripheral tissues in Eomes cKO mice are functionally impaired. The current study reveals the importance of Eomes in the development of NKT1 cells in the thymus and also in their activation and differentiation in peripheral tissues.

## Results

### Eomes affects the development of iNKT cells in the thymus

**Fig. 1:**
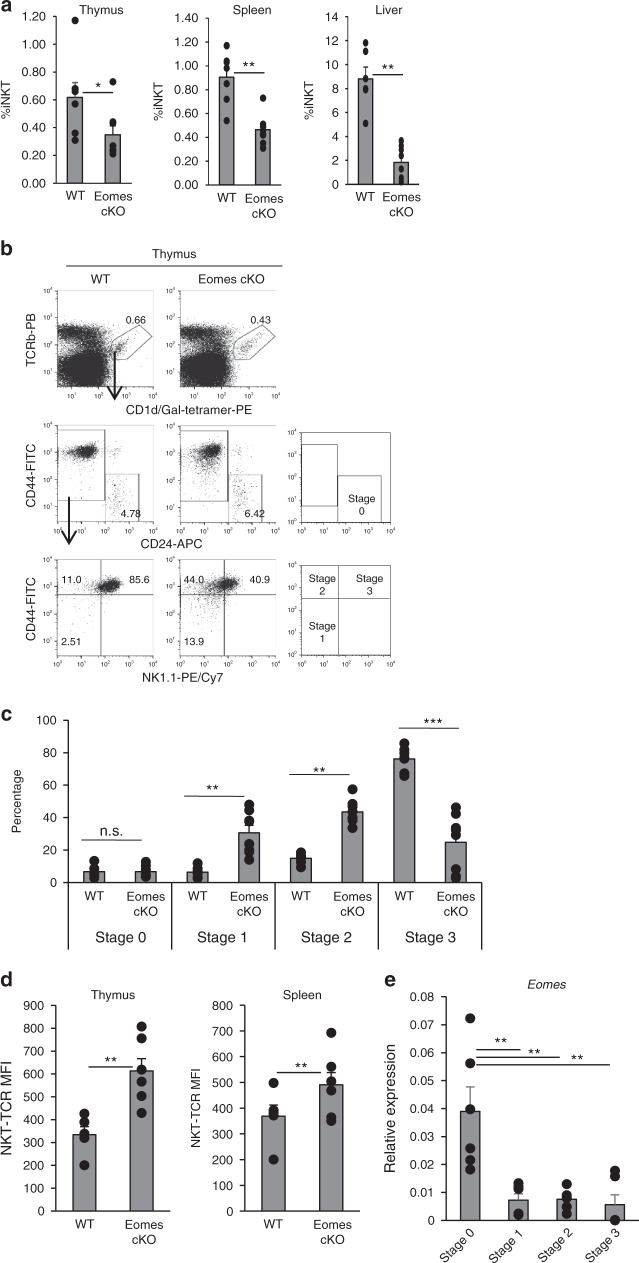
Crucial role of Eomes for iNKT cell terminal differentiation. **a** Percentage of CD1d-tet^+^TCRβ^+^iNKT cells in WT and Eomes cKO thymus, spleen, and liver (*n* = 6–8, mean ± SEM). **b** CD24, NK1.1, and CD44 staining on gated iNKT cells from (**a**). **c** Percentage of stage 0 to 3 iNKT cells from WT and Eomes cKO mice (*n* = 6–8, mean ± SEM). **d** Expression of Vα14TCR in WT and Eomes cKO mice was assessed by flow cytometry as mean fluorescence intensity (MFI). Similar data were obtained from at least three independent experiments. *p < 0.05, **p < 0.01, ***< *p* < 0.001, Mann–Whitney **e** Quantitative PCR analysis of Eomes mRNA in thymic iNKT cells (TCRβ^+^CD1d-tetramer^+^) from WT mice: stage 0 (CD24^+^CD44^−^NK1.1^−^CD69^+^), stage 1 (CD24^−^CD44^−^NK1.1^−^), stage 2 (CD24^−^CD44^+^NK1.1^−^), stage 3 (CD24^−^CD44^+^NK1.1^+^). (*n* = 6, mean ± SEM) **p < 0.01, two-tailed Student’s *t*-test

To investigate the potential role of Eomes in iNKT cell development, we analyzed T cell-specific Eomes-deficient mice, specifically CD4-Cre Eomes^f/f^ (Eomes cKO) mice. Conventional CD4^+^ T cell and CD8^+^ T cell development in the thymus of Eomes cKO mice was unaffected, as assessed by cell numbers and ratios of thymocyte subsets, which were similar to those of wild type mice (Supplementary Fig. 1a). The CD4^+^ to CD8^+^ T cell ratio in the spleen of Eomes cKO mice was also relatively normal (Supplementary Fig. 1a), consistent with a previous report^15^. However, the frequency of iNKT cells in thymus, spleen, and liver of Eomes cKO mice was lower than in WT controls (Fig. 1a). Closer analysis of the thymus revealed that the frequency and absolute number of CD24^−^CD44^−^NK1.1^-^stage 1 and CD44^+^NK1.1^−^ stage 2, iNKT cells were increased, but those of CD44^+^NK1.1^+^ stage 3 iNKT cells were decreased (Fig. 1b, c and Supplementary Fig. 2a). Since thymic iNKT cells contain CD4^+^CD8^−^ single positive and CD4^−^CD8^−^ double negative subsets, we analyzed CD4 and CD8 expression. No change in the ratio of CD4 single positive and CD4^−^CD8^−^ double negative subsets was observed in Eomes cKO mice (Supplementary Fig. 2b). Of note, Eomes cKO mice expressed higher levels of Vα14TCR than WT controls, clearly indicating that the development of iNKT cells in the thymus was impaired (Fig. 1d and Supplementary Fig. 3). These results suggested that the terminal maturation of iNKT cells in the thymus may depend on Eomes expression. Recent studies have suggested that iNKT cell precursors in the thymus receive the strongest TCR signal during selection rather than mature iNKT cells^16,17^. Therefore, we assessed the level of Eomes in NKT stages 0 to 3 in the thymus by quantitative PCR (qPCR). To isolate stage 0 iNKT cells as iNKT precursor cells, we sorted CD1d-tetramer^+^CD24^+^CD44^−^NK1.1^−^CD69^+^ cells. As shown in Fig. 1e, stage 0 iNKT cells showed the highest expression of Eomes compared to the other stages. Thus, the expression of Eomes may be related to TCR signaling in the thymus.

### Loss of Eomes decreases NKT1 cells

**Fig. 2:**
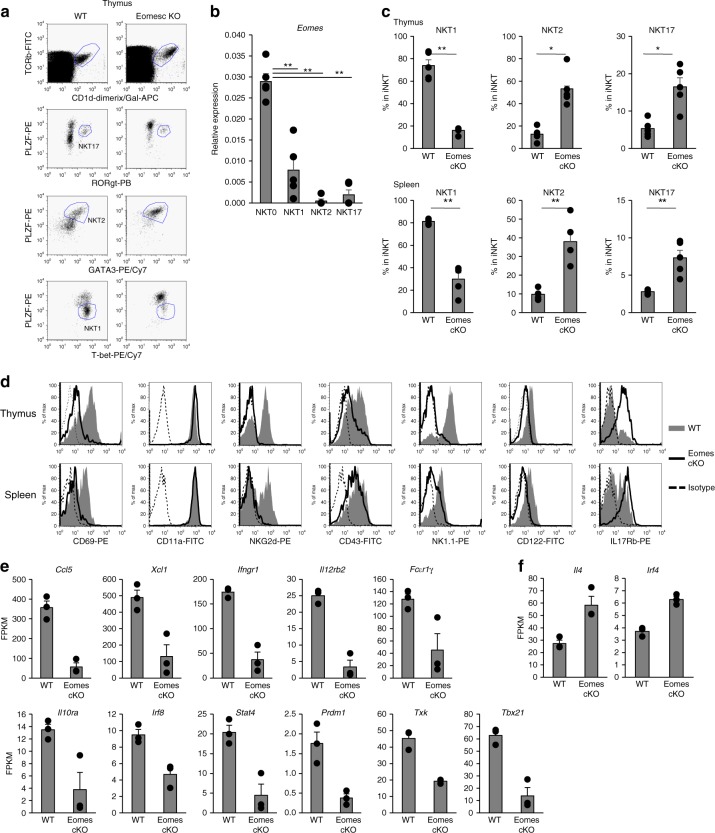
Aberrant expression of molecules affected by Eomes in iNKT terminal maturation and effector function. **a** Expression of PLZF, Rorγt, Gata3, and T-bet by thymic and splenic iNKT from WT and Eomes cKO mice detected by flow cytometry. **b** Quantitative PCR analysis of Eomes mRNA in thymic iNKT cells (TCRβ^+^CD1d-tetramer^+^) from WT mice: NKT0 (CD24^+^CD44^−^NK1.1^−^CD8^−^), NKT1 1(CD24^−^NK1.1^+^CD27^+^CCR6^−^), NKT2 (CD24^−^NK1.1^-^CD27^+^CD4^+^), NKT17 (CD24^−^CD27^−^CD4−CCR6^+^CD103^+^). (*n* = 5, mean ± SEM) **p < 0.01, two-tailed Student’s *t*-test. **c** The percentage of NKT1 (PLZF^dim^T-bet^+^), NKT2(PLZF^high^Gata3^+^), and NKT17(PLZF^+^Rorγt^+^) subsets in thymus and spleen of WT and Eomes cKO mice. (*n* = 5, mean ± SEM). *p < 0.05, **p < 0.01, Mann–Whitney. **d** Representative histograms showing expression of CD69, CD11a, NKG2d, CD43, NK1.1, CD122, and IL17Rb by thymic and splenic iNKT cells from WT and Eomes cKO mice (*n* = 5). Similar data were obtained from at least three independent experiments. **e**, **f** Thymic iNKT cells from WT and Eomes cKO mice were analyzed by RNA-Seq. Expression levels (expressed as FPKM) of selected NKT1 (**e**) or NKT2 (**f**) subset-specific genes of thymic iNKT cells were compared between WT and Eomes cKO mice (*n* = 3, mean ± SEM). (WT vs KO, *p < 0.05, **p < 0.01, ***< *p* < 0.001, two-tailed Student’s *t*-test)

Recently, iNKT cells have been categorized into three lineages based on their master transcription factors. T-bet is critical for iNKT cell terminal maturation and NKT1 generation, whereas Gata3 and Rorγt are crucial transcription factors for NKT2 and NKT17 differentiation, respectively^3,17–20^. We analyzed the expression of several transcription factors related to iNKT cell differentiation, such as T-bet, Gata3, Rorγt as well as the master regulator of all iNKT cells, PLZF (Fig. 2a). We also analyzed the expression level of Eomes in iNKT subsets (NKT0, NKT1, NKT2, and NKT17) in the thymus of WT mice. As shown in Fig. 2b, NKT0 express Eomes at higher levels than other NKT cell subsets. Eomes cKO iNKT cells expressed higher levels of PLZF and Gata3 protein, but decreased levels of T-bet (Fig. 2a–c). Because of the block in the development of iNKT precursors into NKT1 cells, the frequency of NKT2 and NKT17 subsets tended to increase in the thymus. We then assessed the phenotypes of iNKT cells in the thymus and spleen, which should be affected by these transcription factors. NK1.1, NKG2D, CD69, and CD122, a component of the IL-15 receptor that is important for iNKT terminal maturation^15^, were detected in WT mice but their expression was decreased in Eomes cKO iNKT cells (Fig. 2d). By contrast, expression of IL-17Rb, which is an IL-25 receptor that defines the IL-4 producing capacity of iNKT cells^21^, was substantially increased in Eomes cKO iNKT cells (Fig. 2d).

To better understand Eomes-mediated regulation of the differentiation of iNKT cells, we examined the expression of cytokines, chemokines, and transcription factors in thymic iNKT cells by RNA sequencing (RNA-seq). Compared to WT mice, Eomes cKO mice showed decreased expression of NKT1-related chemokines (*Ccl5* and *Xcl1*), cytokine receptors (*Ifnγr1, Il12rb2, Il10ra*), signaling adaptor molecules (*FcɛR1γ*) and transcription factors and signaling pathway molecules (*Irf8, Stat4, Prdm1, Txk* and *Tbx21*), but increased expression of NKT2-related molecules such as *Il4* and *Irf4* (Fig. 2e, f). These results indicated that Eomes regulates not only the differentiation, but also the function of NKT1 cells in the thymus.

### Eomes alters IFN-γ production in iNKT cells

**Fig. 3:**
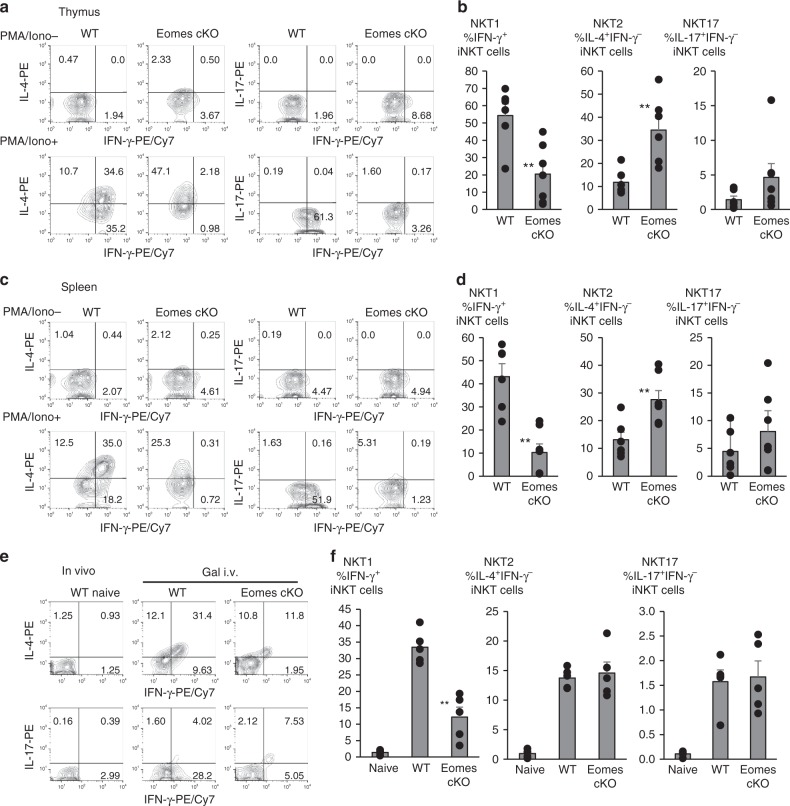
Suppression of the differentiation of IFN-γ producing iNKT cells in Eomes cKO. **a**, **b** Percentage of IFN-γ, IL-4, and IL-17A production by thymic iNKT cells stimulated with PMA and Ionomycin (Iono) in WT and Eomes cKO mice. (*n* = 6–7, mean ± SEM) **c**, **d** As in (**a**, **b**), percentage of splenic iNKT cells positive for the indicated cytokines in WT and Eomes cKO mice. (*n* = 6–7, mean ± SEM) **e**, **f** Percentage of splenic iNKT cells positive for IFN-γ, IL-4, and IL-17A production in WT or Eomes cKO 2 h after i.v. administration of α-GalCer (1 μg/mouse) (*n* = 5–6; data are shown as mean ± SEM). Similar data were obtained from at least three independent experiments. **p < 0.01, Mann–Whitney

The presence of iNKT cells in Eomes cKO mice allowed us to examine how Eomes deficiency may affect iNKT cell effector function. NKT1 cells can produce IFN-γ and IL-4, whereas NKT2 cells produce IL-4 but not IFN-γ. NKT17 cells secrete IL-17, but not IFN-γ. Following in vitro stimulation with PMA plus ionomycin for 6 h, WT iNKT cells predominantly produced IFN-γ and IL-4, but minimally produced IL-17 (Fig. 3a, b). In contrast, there was a severe reduction in NKT1 cells capable of producing both IFN-γ and IL-4 in the Eomes cKO, while the frequency of NKT2 cells producing only IL-4 increased dramatically (Fig. 3a, b). Similar to thymocytes, there were fewer iNKT cells in Eomes cKO spleen that produced both IFN-γ and IL-4 than in WT controls (Fig. 3c, d). Compared to NKT1 cells, NKT17 cells appeared to increase in Eomes-deficient mice (Fig. 3b–d). These data might suggest that NKT2 and NKT17 cells are selectively increased in Eomes cKO mice, but that is not actually the case. The observed increase in NKT2 and NKT17 cells is caused by the decrease of NKT1 cells.

Next, we examined the in vivo response of iNKT cells in Eomes cKO mice. The mice were injected i.v. with α-GalCer and splenic iNKT cells were analyzed by intracellular cytokine staining 2 h later. Consistent with the in vitro observations, fewer Eomes cKO iNKT cells produced both IFN-γ and IL-4 than WT iNKT cells (Fig. 3e, f). Together, the regulation of IL-4 and IFN-γ by Eomes in iNKT cells was developmentally programmed, i.e., the differentiation of NKT1, but not NKT2 and NKT17 cells was blocked by Eomes deficiency. These observations are consistent with a block at stage 3 in Eomes cKO mice.

### Cell-intrinsic mechanism in iNKT development by Eomes

**Fig. 4:**
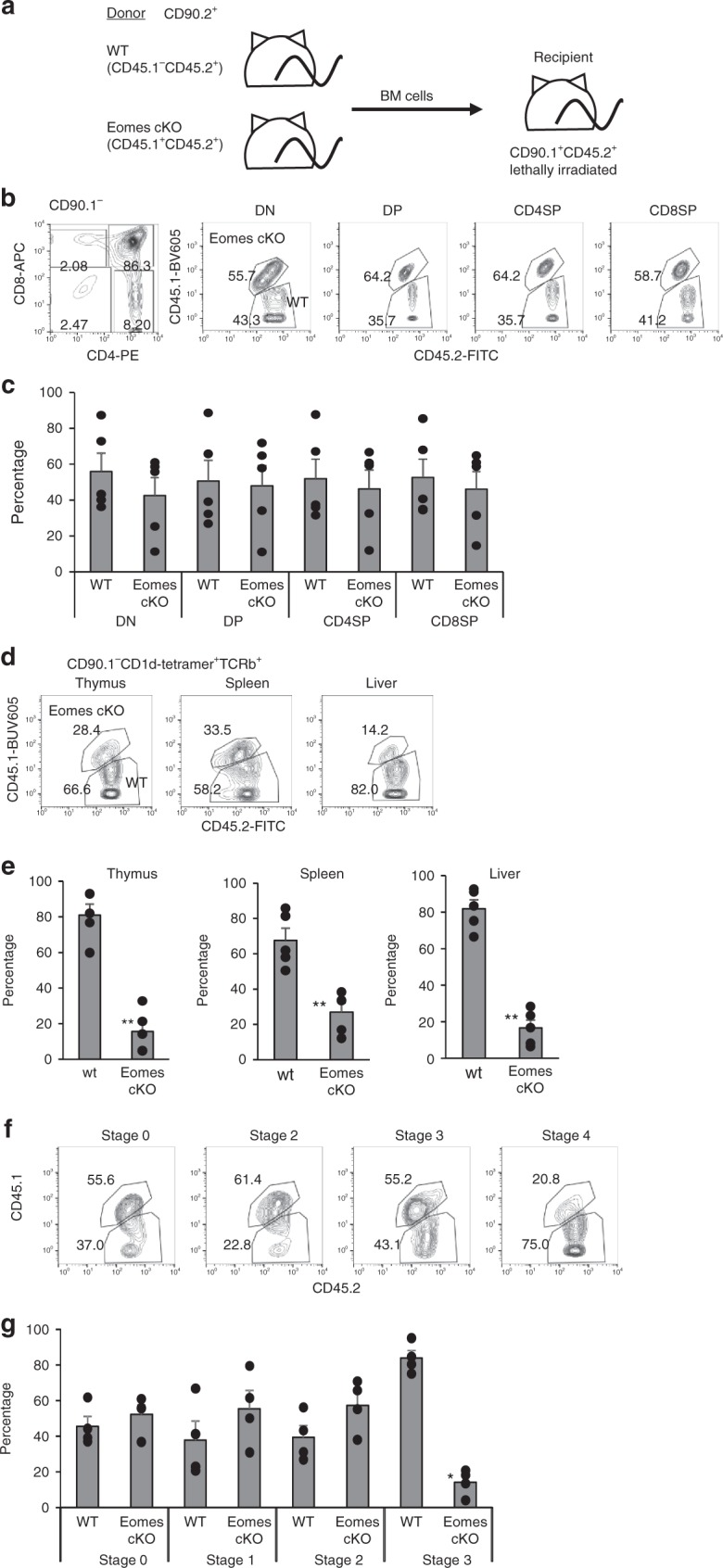
Regulation of NKT1 cell development by Eomes is cell-intrinsic. **a** Experimental set-up of the BM chimera experiment. CD90.1^+^CD45.1^−^CD45.2^+^ recipient B6.PL mice were lethally irradiated and transplanted i.v. with a 1:1 mixture of CD90.2^+^ CD45.1^−^CD45.2^+^ B6 BM together with BM from CD90.2^+^CD45.1^+^CD45.2^+^Eomes-cKO mice. Recipient mice were sacrificed 8 w after transplantation and iNKT cells in thymus, spleen, liver, and lung were analyzed for the expression of CD45.1 and CD45.2. **b** Representative dot plots show CD4 and CD8 expression by thymocytes (left) and the indicated subsets (double negative, double-positive and single positive) of chimeric mice (right). **c** The percentage of each subset generated from WT and KO BM cells. (*n* = 4, mean ± SEM). **d** Representative dot plots show CD45.1 and CD45.2 expression by iNKT cells in the thymus, spleen, liver, and lung of the chimeric mice. **e** The percentage of iNKT cells in each organ generated from WT and KO BM cells. (*n* = 4, mean ± SEM). **f** Representative dot plots show CD45.1 and CD45.2 expression by iNKT cells at different development stages (Stage 0–3) within the thymus of the chimeric mice (upper). **g** The percentage of iNKT cells at each stage generated from WT and KO BM cells. (*n* = 4, mean ± SEM). Data are combined from two independent experiments. *p < 0.05, **p < 0.01, Mann–Whitney

To address whether Eomes regulated the thymic differentiation of iNKT cells in a cell-intrinsic manner, we reconstituted lethally irradiated CD45.2^+^CD90.1^+^ wild type recipient mice with WT CD45.2^+^CD90.2^+^ BM cells and Eomes cKO CD45.1^+^CD45.2^+^ CD90.2^+^ BM cells at a ratio of 1:1 (Fig. 4a). After 8 weeks engraftment, CD4^−^CD8^−^ double negative, double-positive and single positive thymocytes developed in the recipient mice (Fig. 4b). The WT to KO ratios of double negative, double-positive and single positive thymocytes ranged from 0.8–1.2, close to the original 1:1 ratio (Fig. 4b, c), suggesting that Eomes is not essential for αβT cell maturation in the thymus, even in a competitive environment. We verified that iNKT cells were also reconstituted in the mixed chimeric mice (Fig. 4d–g), and compared the ability of each donor population to reconstitute the CD1d-Tet^+^ iNKT cells in the thymus, spleen, liver, and lung. When we focused on the reconstituted iNKT cells in the thymus, we found fewer iNKT cells of Eomes cKO origin than of wild type origin, the WT to KO ratios of iNKT cells were more than 2 (Fig. 4d, e). Similar results were also observed with splenocytes and liver mononuclear cells (MNCs) from the chimeric mice (Fig. 4d, e) Furthermore, we analyzed the stages of iNKT cells and found a similar frequency of Eomes-deficient and wild-type derived Stage 0-Stage 2 cells, but a greatly decreased frequency of Eomes-deficient stage 3 iNKT cells (Fig. 4f, g). These findings suggested an essential and cell-intrinsic role for Eomes in the terminal maturation and maintenance of iNKT cells in the thymus.

### TCR signaling induces Eomes expression in thymic iNKT cells

**Fig. 5:**
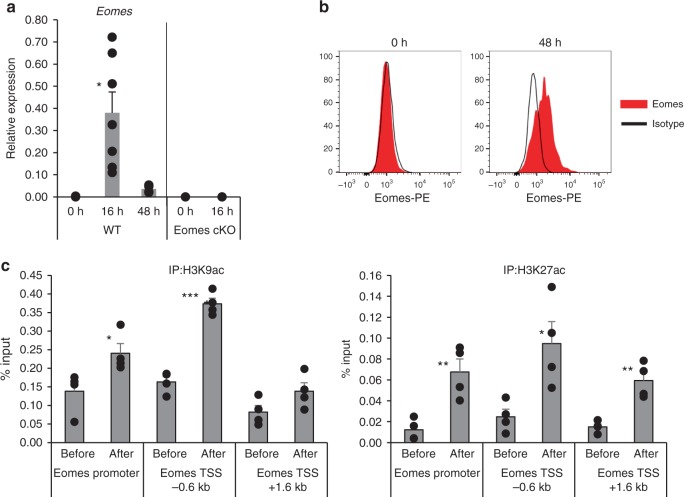
Transient expression of Eomes by iNKT cells is epigenetically regulated. **a** Kinetics of Eomes mRNA expression in non-activated (0 h) and activated (16, 48 h) thymic iNKT cells. Total thymic iNKT cells from WT mice were stimulated with anti-CD3 plus anti-CD28 mAbs for the indicated periods and the levels of Eomes transcripts were determined by qPCR. Sorted thymic iNKT cells from Eomes cKO were used as a negative control. (*n* = 3–7, mean ± SEM). Data are combined from four independent experiments. **b** Expression of Eomes in non-activated (0 h) and activated (48 h) thymic iNKT cells assessed by flow cytometry. (Red, Eomes; Black, isotype) (*n* = 3) **c** Chip analysis for H3K9ac and H3K27ac modifications at the Eomes locus in non-activated and activated thymic iNKT cells (*n* = 4, mean ± SEM). Data are summarized from three independent experiments. *p < 0.05, **p < 0.01, ***< *p* < 0.001, Mann–Whitney

As shown in Fig. 1e, the expression of *Eomes* in iNKT cells in the steady state is quite low. Next, we examined whether Eomes in iNKT cells can be upregulated by TCR stimulation. For this, iNKT cells were sorted from thymus and stimulated with anti-CD3 and anti-CD28 mAbs. We found that the expression of Eomes mRNA was upregulated at 16 h after TCR stimulation, but not in Eomes cKO mice (Fig. 5a) and was also elevated at the protein level 48 h after the stimulation (Fig. 5b). These results indicate that expression of Eomes can be induced upon TCR stimulation of iNKT cells. Thus, Eomes shows a unique expression pattern, with little expressed in the steady state. It is expressed transiently, but apparently only in the early activation phase. We hypothesized that such transient expression should be regulated epigenetically and therefore evaluated histone acetylation (ac), an epigenetic modification associated with accessible chromatin structure and active transcription. As shown in Fig. 5c, both H3K9ac and H3K27ac were increased at the *Eomes* locus in activated iNKT cells.

### Eomes regulates the activation of iNKT cells in periphery

**Fig. 6:**
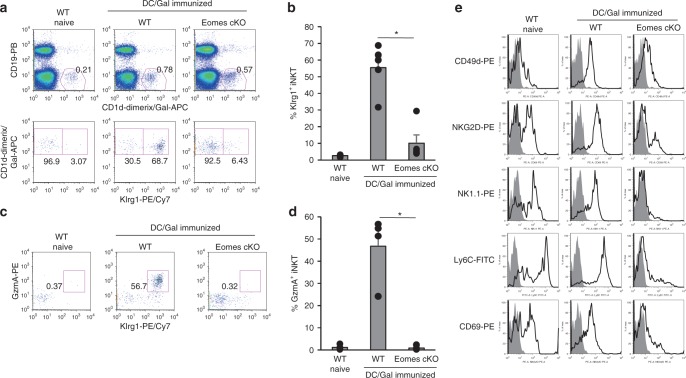
Eomes-deficient iNKT cells fail to differentiate into Klrg1^+^ iNKT cells after DC/Gal immunization. WT and Eomes cKO mice were immunized with DC/Gal. Four weeks later, lung iNKT cells were analyzed by flow cytometry. **a**, **b** The expression and frequency of lung Klrg1^+^ iNKT cells from naïve WT or DC/Gal-immunized WT or Eomes cKO mice. (*n* = 5) **c**, **d** The expression and frequency of lung granzyme A^+^ iNKT cells (*n* = 5, mean ± SEM) **e** Expression of the indicated proteins by lung iNKT cells from WT naïve mice or DC/Gal-immunized, WT or Eomes cKO mice. (*n* = 5) Similar data were obtained from at least three independent experiments. *p < 0.05, Mann–Whitney

After showing that Eomes regulates the differentiation and function of NKT1 cells in the thymus, we next examined its effect on the activation phase of iNKT cells in peripheral tissues, which was unknown. Lee et al. reported that NKT2 and NKT17 cells predominate in lung in the steady state^22^. However, we previously reported that the number of NKT1-polarized Klrg1^+^ iNKT cells markedly increased in the lung of WT mice after vaccination with DC/Gal^7^. We also observed dominant expression of *Eomes* in Klrg1^+^ iNKT cells, but not in naïve iNKT cells. As previously demonstrated, we verified the expression of Klrg1 and granzyme A (Fig. 6a–d) as well as NK1.1, CD49d, NKG2D, Ly6C, and CD69 (Fig. 6e) by WT Klrg1^+^ iNKT cells in the lung after DC/Gal immunization. By contrast, in the DC/Gal-injected Eomes cKO mice, the generation of Klrg1^+^gzmA^+^ lung iNKT cells was inhibited (Fig. 6a–d*)*, as was the expression of CD49d, NKG2D, NK1.1 and Ly6C (Fig. 6e). These results indicate that the peripheral differentiation of iNKT cells triggered by iNKT ligand and TCR signaling is also regulated by Eomes. And, they also suggest that NKT1 cells may require TCR signaling during their differentiation into memory-like iNKT cells.

**Fig. 7:**
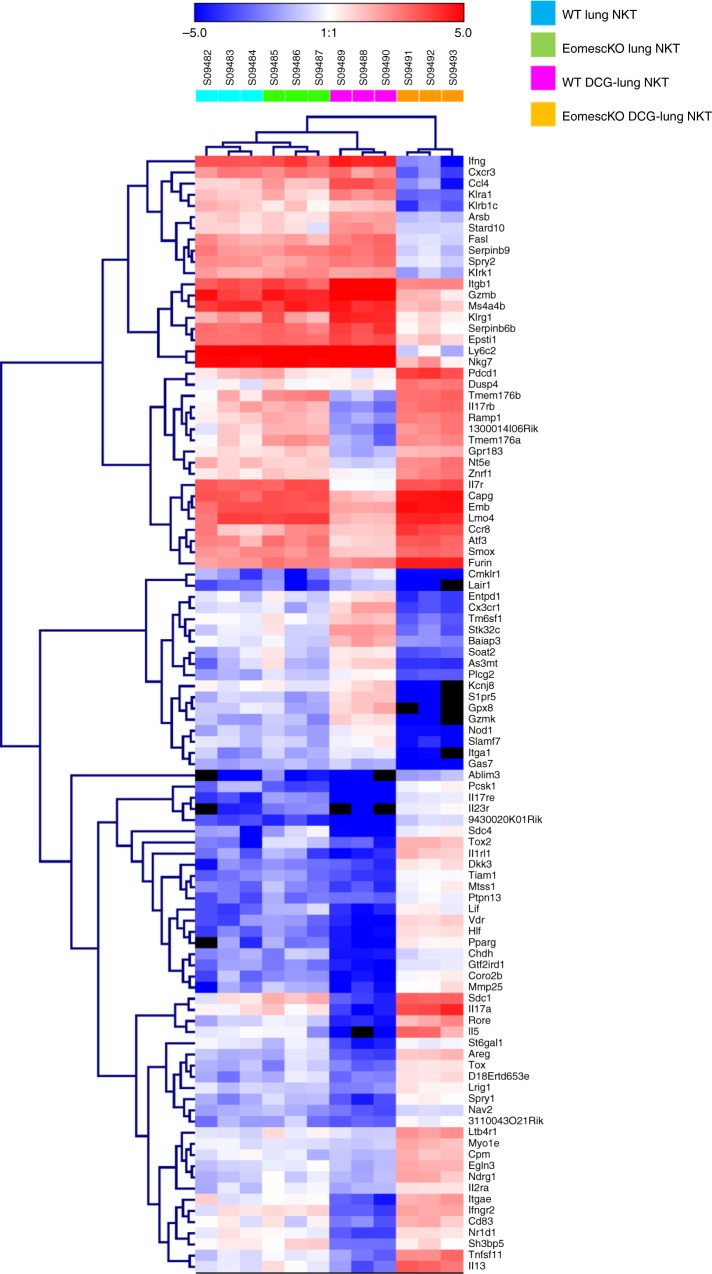
Differentially expressed genes between iNKT cells from naive or DC/Gal primed WT and Eomes cKO mice. WT and Eomes cKO mice were immunized i.v. with DC/Gal. One week later, lung iNKT cells were sorted and analyzed by RNA-Seq. The differential expression analysis was performed using an R based package, EdgeR with the raw counts from RNA-seq data of lung iNKT cells from DC/Gal primed WT and Eomes cKO mice (*n* = 3). Clustering data of top 100 differentially expressed genes are shown in a heat map format with their gene symbols

**Fig. 8:**
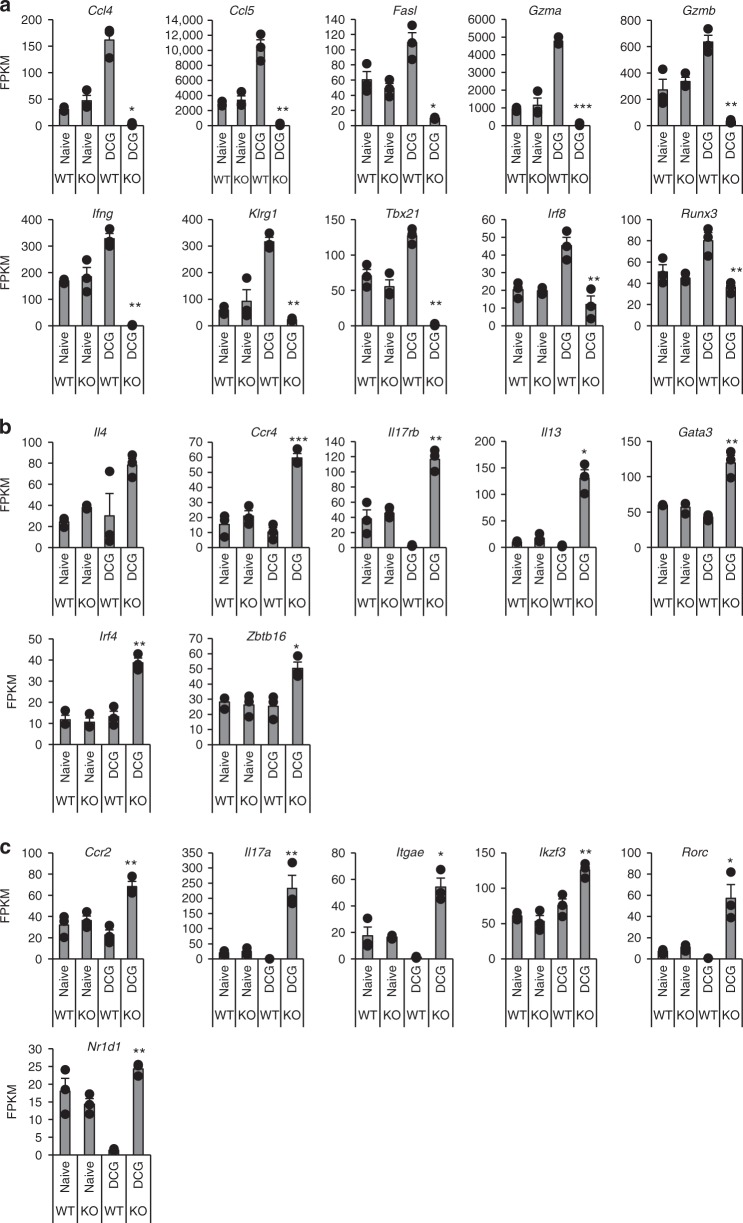
Comparison of RNA profiles of lung iNKT cells from naive or DC/Gal primed WT and Eomes cKO mice. WT and Eomes cKO mice were immunized i.v. with DC/Gal. One week later, lung iNKT cells were sorted and analyzed by RNA-Seq. **a**–**c** Expression levels (expressed as FPKM) from selected NKT1 (**a**) or NKT2 (**b**) or NKT17 (**c**) subset-specific genes of the lung iNKT cells between naïve or DC/Gal-primed WT and Eomes cKO mice were compared. (*n* = 3, mean ± SEM). (WT DC/Gal vs cKO DC/Gal, *p < 0.05, **p < 0.01, ***< *p* < 0.001, two-tailed Student’s *t*-test)

To further characterize the gene-expression program in iNKT cell subsets in the lung from WT and Eomes cKO mice given DC/Gal, we performed pairwise comparison of bulk RNA-seq (Figs 7 and 8). In the steady state, we found no difference in iNKT cells from WT and Eomes cKO mice. Given that NKT2 and NKT17 are dominant in the lung in the steady state^22^, this result was predictable. In contrast, we observed obvious differences in function-related molecules, such as effector molecules, cytokines, and chemokines, by the transcriptome analysis of activated iNKT cells of Eomes cKO and WT mice after immunization with DC/Gal (Fig. 7). Transcripts encoding NKT1 effector molecules, e.g., *GzmA, GzmB*, and *Fasl*, were more highly expressed in iNKT from WT mice injected with DC/Gal than in untreated or DC/Gal-injected Eomes cKO mice (Fig. 8a). The iNKT cells in DC/Gal-injected Eomes cKO mice showed high level expression of transcripts encoding NKT2 or NKT17 type cytokines and chemokines (*Ccr2, Ccr4, Il17rb, Il13, Il17a*) and signaling pathway molecules (*Gata3, Irf4, Zbtb16, Ikzf3, Rorc*, and *Nr1d1*) (Fig. 8b, c). Thus, when DC/Gal was administered, NKT1 cells were increased in WT mice, whereas NKT2 and NKT17 cells were increased in Eomes cKO mice. The NKT1 transcript signatures are therefore reciprocally regulated by Eomes with transcripts of NKT2 and NKT17 cells during their activation in peripheral organs.

## Discussion

Eomes is well known to regulate the differentiation of certain effector cells, such as CD4^+^ and CD8^+^ T cells and NK cells, and is also involved in memory T cell induction^23^. In the current study, we demonstrated an impact of Eomes on iNKT differentiation in the thymus and on the activation program in peripheral iNKT cells. Eomes controls the differentiation from the iNKT precursor to NKT1 cells in the thymus. In addition, Eomes also regulates NKT1 cell differentiation in the peripheral tissues. We previously showed that after activation with the iNKT ligand-loaded DCs in the peripheral tissues, some activated iNKT cells become skewed toward long-term memory type NKT1 cells. However, they fail to do so in the Eomes cKO mice.

Three recent studies analyzed global gene expression profiles of thymic NKT1, NKT2, and NKT17 cells and their enhancer landscape and revealed that the three subsets are transcriptionally distinct^24–26^. Engel et al. identified the enhancer profiles of each NKT subset by H3K27ac Chip-Seq^25^. They found that enhancers in NKT1, NKT2, and NKT17 cells that had increased H3K27ac showed enrichment at binding motifs for T-bet, Gata family and Rorγt, respectively. With regard to Eomes, they described enrichment at Eomes binding motifs in the enhancers in NKT1 cells. However, because expression of Eomes in iNKT cells was low, they suspected that this finding might reflect the activity of another transcription factor with a similar motif^25^. It is true that Eomes expression is quite low in iNKT cells in the steady state (Figs 1, 5). Some studies previously proposed that stage 0 iNKT cells received a stronger TCR signal during selection rather than mature iNKT cells using Nur77^GFP^ mice, which upregulate GFP in response to Ag receptor but not inflammatory signals^16,17^. Indeed, we showed that among thymic iNKT cells, the highest expression of Eomes was in stage 0 iNKT cells and then it decreased in stage 1–3 iNKT cells. We also showed that upregulation of Eomes in the thymic iNKT cells was induced by TCR stimulation in vitro (Fig. 5). Both of these findings suggest that *Eomes* upregulation during iNKT cell development in thymus may be induced by TCR signaling.

The relationship between Eomes expression and the acquisition of NKT1 cell phenotype and function during the development of iNKT cells in the thymus is unclear. It is known that different NKT cell subsets express different levels of TCR^26,27^. In addition to such TCR signal strength, transcription factors, epigenetic changes, and cytokines may play a role in the development of iNKT subsets in the thymus. CD122 is a shared subunit of IL-2 and IL-15 receptors, and responsiveness to IL-15 is essential for the final maturation into Stage 3 iNKT cells^6^. Also, after receiving a signal via CD122-IL-15, the micro-RNA let-7 is upregulated in iNKT precursor cells and that inhibits PLZF, thus skewing NKT1 differentiation^28^. It thus seems possible that IL-15-CD122 may help in the transit from iNKT cell precursor toward NKT1 differentiation. By transcriptome analysis of thymic iNKT cells from WT and Eomes cKO mice, we found that expression of NKT1-specific molecules, i.e., cytokine and chemokine receptors as well as cytokines, chemokines and transcription factors, was decreased, whereas NKT2- and NKT17-related molecules were upregulated (Fig. 2e, f)^6,26,27,29^. These may be affected by the altered ratio of NKT1, 2 and 17 cells. However, it was previously reported that CD122 expression is a direct target of Eomes in T cells^9^. There thus remains the possibility of direct regulation of CD122 by Eomes in iNKT cells. In elucidating an interaction between Eomes and CD122 in the development of thymic NKT1 cells, further studies of whether it interacts directly or indirectly through other cytokines or transcription factors will be required.

**Fig. 9:**
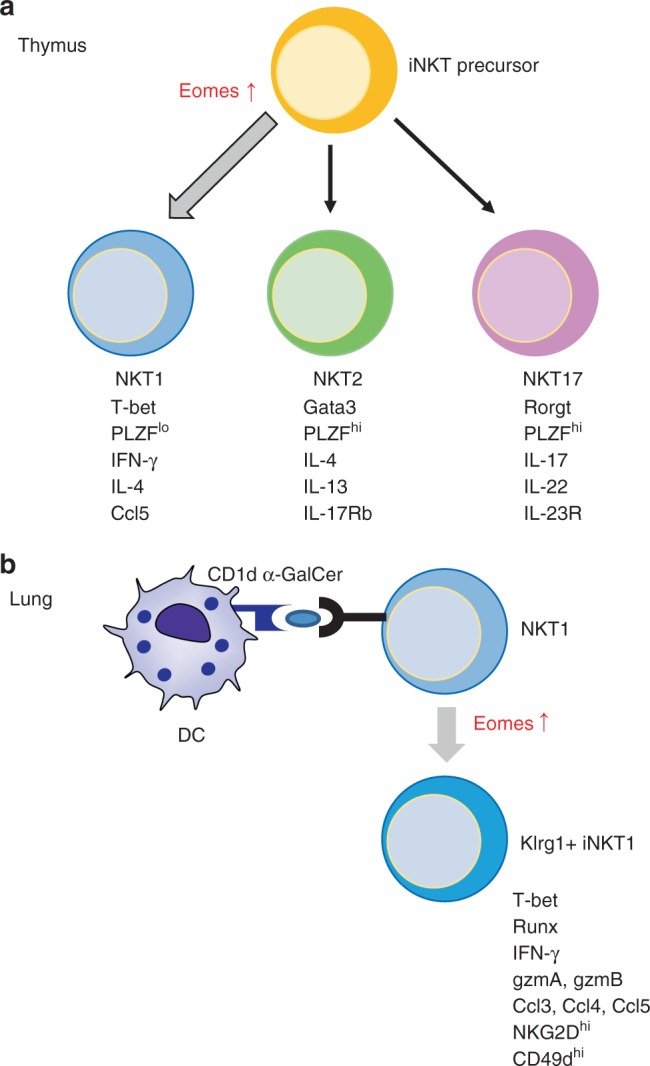
The model depicting the role of Eomes during iNKT development in the thymus (**a**) and differentiation in the lung (**b**) (see Discussion for details)

Klrg1^+^ iNKT cells in the lung were generated and maintained for the long term after administration of DC/Gal (Fig. 9)^7^. These cells displayed NKT1-like cytokine and chemokine profiles (predominant IFN-γ, CCL3, -4, and -5 production) and prolonged survival as memory-like iNKT cells expressing Tbx21, Runx3, and Eomes. The molecular mechanism by which the Klrg1^+^ iNKT population is generated after administration of DC/Gal is not yet clearly defined. As previously reported, NKT2 cells localize inside the blood vasculature and NKT17 cells reside within the lung parenchyma, whereas NKT1 cells were detected in both vasculature and parenchyma^22^. Intravenously administered DC/Gal can be trapped and retained in the lung for at least 24 h^7^. Therefore, these intravascular iNKT cells in the lung could interact directly with DC/Gal, thereby receiving TCR as well as cytokine signaling. In the current study, we demonstrated that Klrg1^+^ iNKT cells are not generated in Eomes cKO mice, even after administration of DC/Gal. These results clearly imply that generation of Klrg1^+^ iNKT cells requires Eomes expression (Fig. 9). Given that Klrg1^+^ iNKT cells express Eomes, TCR signaling and cytokine signaling may reactivate Eomes expression and switch the peripheral differentiation of NKT1 cells into the Klrg1^+^ iNKT cell pathway. Taken together, Eomes is crucial, not only for the development of iNKT cells in the thymus, but also for their further differentiation in the periphery.

## Methods

### Mice

C57BL/6 mice and B6.PL were purchased from CLEA Japan and Jackson Laboratory respectively. Eomes^Exon1-f/Exon1-f^-CD4Cre^*+*^ mice (Eomes cKO) were kindly provided by Dr. T. Nakayama (Chiba University) and Dr. Steven L. Reiner (Columbia University) and backcrossed to C57BL/6 mice for 12 generations. Mice 6–8 wk of age were used for experiments. All the mice were maintained under specific pathogen-free conditions and all procedures were performed in compliance with the protocols approved by the Institutional Animal Care Committee at RIKEN. Mixed-bone marrow (BM) chimeras containing wild-type (CD45.1^−^CD45.2^+^CD90.2^+^) and Eomes cKO BM (CD45.1^+^CD45.2^+^CD90.2^+^) at a ratio of 1:1 were generated by lethal irradiation of B6.PL recipients (CD45.1^−^CD45.2^+^CD90.2^+^) (two doses of 5 Gy each), followed by reconstitution by intravenous injection of 1 × 10^7^ donor BM cells. Mixed BM transfer mice were analyzed 8 weeks after transplantation.

### Cell preparation

Thymocytes and splenocytes were obtained by pressing thymus and spleen through a 70 μm cell strainer and erythrocytes were lysed with ACK lysing buffer (GIBCO) followed by two washes in RPMI. For isolation of lung and liver MNCs, the tissues were digested with collagenase D (Roche) and then layered on Percoll gradients (40/60%) (Amersham Pharmacia Biotec) and centrifuged for 20 min at 900 × *g*. BM-derived DCs were generated in the presence of GM-CSF and pulsed with 100 ng ml^−1^ α-GalCer for 48 h from day 6 and matured by LPS as previously described^30^. For RNA isolation from thymic iNKT cells, thymocytes were enriched for iNKT cells by negative selection using anti-CD8 micro beads (Milteny Biotec) and MACS magnet and LS columns. The remaining cells were stained with CD1d-dimers loaded with α-GalCer followed by anti-Mouse IgG-PE and anti-TCR-β-PB and Lived/Dead Aqua. Then, TCRβ^int^ CD1d-dimer/Gal^+^ cells (iNKT cells) were sorted with a FACSAria (BD) (purity, >95%). For RNA isolation from thymic iNKT cell stages or subsets, thymus cell suspensions were pooled from 6 WT mice. Each iNKT cell (CD1d-tet/Gal^+^TCRb^int^) stage was sorted as followed: stage 0 (CD8^−^CD24^+^CD44^−^NK1.1^−^CD69^+^), stage 1 (CD24^−^CD44^−^NK1.1^−^), stage 2 (CD24^−^CD44^+^NK1.1^−^), stage 3 (CD24^−^CD44^+^NK1.1^+^) **(**Supplementary Fig. 4a). iNKT subsets (CD1d-tet/Gal^+^TCRb^int^) were sorted as followed:^25^ NKT0 (CD8^−^CD24^+^CD44^−^NK1.1^−^), NKT1(CD24^−^NK1.1^+^CD27^+^CCR6^−^), NKT2 (CD24^−^NK1.1^−^CD27^+^CD4^+^), NKT17 (CD24^−^CD27^−^CD4^−^CCR6^+^CD103^+^) (Supplementary Fig. 4b). For RNA isolation from lung iNKT cells, lung MNCs were stained with CD1d-dimers loaded with α-GalCer, followed by anti-Mouse IgG-PE and anti-CD19-PB and Lived/Dead Aqua. Then, CD19^−^ CD1d-dimer/Gal^+^ cells (iNKT cells) were sorted with a FACSAria (BD).

### Flow cytometry

The following monoclonal antibodies (mAbs) were purchased from BD Bioscience, BioLegend, or e-Bioscience: anti-CD4 (GK1.5), anti-CD8 (53.67), anti-CD16/32 (93), anti-CD19 (6D5), anti-CD24 (M1/19), anti-CD27(LG.3A10), anti-TCRβ (H57-597), anti-CD43 (S7), anti-CD44 (1M7), anti-CD11a (M17/4), anti-CD69 (H1.2F3), anti-NKG2D (CX5), anti-NK1.1 (PK136), anti-CD103 (2E7), anti-CCR6 (29-2L17), anti-CD122 (TM-β1), anti-CD45.1 (A20), anti-CD45.2 (104), anti-EGR2 (erongr2), anti-T-bet (eBio4B10), anti-Gata3 (TWAJ), anti-Rorγ (B2D), anti-PLZF (9E12), anti-Eomes (Dan11mag), anti-IFN-γ (XMG1.2), anti-IL-4 (11B11), anti-IL-17 (TC11-18H10), anti-IL-17RB(9B10) and CD1d-dimers. CD1d-tetramers were purchased from MBL. Fixable Aqua Dead Cell stain kit (Invitrogen) was used to eliminate dead cells. Intracellular staining for transcription factors was performed using the eBioscience Foxp3 Staining Buffer Kit. Intracellular staining for cytokines was performed using the BD Cytofix/Cytoperm™ Kit. For analysis, a FACSCalibur, Canto II or LSRFortessa X-20 instrument and the CELLQuest or FACSDiva (BD Biosciences) or FlowJo software (v10.3B2) packages were used.

### Activation of iNKT cells

For in vivo activation, mice were administered 1 μg α-GalCer (Funakoshi) by i.v. injection. Two hours later, splenocytes were analyzed for cytokine production. For in vitro activation, 5 × 10^6^ splenocytes or thymocytes were stimulated with 50 ng/ml PMA (SIGMA) and 500 ng/ml ionomycin (SIGMA) in the presence of GolgiPlug (BD) for 6 h. In some experiments, sorted thymic iNKT cells were stimulated with 10 μg/ml immobilized anti-CD3 mAb and 2 μg/ml soluble anti-CD28 mAb for 16 h or 48 h.

### Quantitative PCR

Cells were lysed in TRIzol LS (Invitrogen) and the RNA precipitation was done with 20 μg RNAase-free glycogen (Invitrogen) and 500 μl Isopropanol (SIGMA). The RNA pellets were washed with 1 ml of 70% cold ethanol. Extracted RNA was quantified with Quant-iT™ RiboGreen® RNA Assay Kit (Thermo Fisher Scientific). cDNA synthesis was performed with the Ovation qPCR system (Nugen) from 10 ng RNA as a template. When RNA concentration was lower than 2.5 ng/μg, SMART-Seq v4 Ultra Low Input RNA kit (Takara Bio) was used for cDNA synthesis from 1 ng RNA as a template.

Quantitative PCR was performed using a StepOne Plus instrument (Applied Biosystems) with FastStart Universal Probe Master mix (Roche). The following primer pair and probes were used: Eomes (forward) 5′-accggcaccaaactgaga-3′, (reverse) 5′-aagctcaagaaaggaaacatgc-3′, UPL probe #9. HPRT1 (forward) 5′-cctcctcagaccgcttttt-3′, (reverse) 5′-aacctggttcatcatcgctaa-3′, UPL probe #95. Relative gene expression was calculated using the ΔΔCT method with the expression of HPRT1 as the internal control.

### Next-generation sequencing (RNA-seq)

RNA was prepared with the RNeasy Plus Micro Kit (Qiagen). RNA-seq library samples were prepared using an NEBNext Ultra RNA Library Prep Kit for Illumina (New England BioLabs). The sequencing was carried out using a HiSeq1500 equipment (Illumina). The sequence reads were mapped to the mouse genome (NCBI version 37) using TopHat2 (version 2.0.8) and botwie2 (version 2.1.0) with default parameters, and gene annotation was provided by NCBI. Transcript abundances were estimated using Cufflinks (version 2.1.1). Cufflinks was run with the same reference annotation with TopHat2 to generate FPKM (fragments per kilobase per million mapped reads) values for known gene models.

### Chromatin immunoprecipitation (ChIP) and ChIP-qPCR

ChIP was as previously described^31^. Sorted iNKT cells were fixed with 1% formaldehyde for 10 min at RT, followed by quenching of formaldehyde with 125 mM glycine for 5 min at RT. Fixed iNKT cells were frozen by liquid nitrogen, and stored in –80 °C. Cross-linked genomic DNA was lysed in lysis buffer [50 mM Tris-HCl (pH 8.0), 10 mM EDTA (pH 8.0), 1% SDS, and 1 × cOmplete Protease Inhibitor Cocktail (Roche)], and sonicated with a Picoruptor (Diagenode), 15 cycles of sonication (30 s) and interval (30 s) at 4 °C. A complex of Dynabeads Protein G (50 μl, ThermoFisher Scientific) and antibody (5 μg) was formed in 0.02% Tween 20 in PBS for 6 h at 4 °C. Then, ChIP was performed in IP buffer [20 mM Tris-HCl (pH 8.0), 2 mM EDTA (pH 8.0), 1% Triton-X100, 150 mM NaCl, and 1 × cOmplete Protease Inhibitor Cocktail] for 21 h at 4 °C. ChIP samples were washed with wash buffer (50 mM HEPES-KOH (pH 7.5), 500 mM LiCl, 1 mM EDTA, 0.7% sodium deoxycholate, 1% Nonidet P-40) 5 times, followed by a wash with TE buffer (Nippongene) twice. DNA was eluted in elution buffer [50 mM Tris-HCl (pH 8.0), 10 mM EDTA (pH 8.0), 1% SDS, and 40 μg Proteinase K (Wako)] for 16 h at 65 °C. DNA was purified with MinElute PCR Purification Kit (Qiagen). Antibodies for ChIP were anti-H3K9ac (Millipore, #06–599), anti-H3K27ac (Active Motif, #39133), and normal rabbit IgG (Millipore, #12–370). Enrichment was analyzed with qPCR (see above) using the standard curve method. Gene-specific forward and reverse primers are described in Supplementary Table 1.

### Statistical analysis

Statistical analysis was performed using StatMate (Ver 5.01). The *F*-test was used to tests for normal distribution of the data. Differences were analyzed using a two-tailed Student’s *t*-test or Mann–Whitney *U*-test. All data are presented as mean ± SEM. *p* < 0.05 was considered statistically significant.

### Reporting summary

Further information on experimental design is available in the Nature Research Reporting Summary linked to this article.

## Supplementary information

Reporting Summary

Description of Additional Supplementary Files

Supplementary information

Supplementary Data 1

## Data Availability

RNA-seq data that support the findings of this study have been deposited in the Gene Expression Omnibus (GEO) database with accession codes GSE128069. Source data underlying the graphs presented in the main figures is available in Supplementary Data 1.
