# Supplementary material for: Eomes transcription factor is required for the development and differentiation of invariant NKT cells
Source: Commun Biol. 2019 Apr 29;2:150. doi: 10.1038/s42003-019-0389-3 (PMC6488575; doi:10.1038/s42003-019-0389-3)
Supplement: Supplementary file 2 — Description of Additional Supplementary Files [file 42003_2019_389_MOESM2_ESM.docx]

**Description of additional supplementary items**

**Supplementary Data 1** Source data underlying the graph presented in Figures
