# Supplementary material for: Eomes transcription factor is required for the development and differentiation of invariant NKT cells
Source: Commun Biol. 2019 Apr 29;2:150. doi: 10.1038/s42003-019-0389-3 (PMC6488575; doi:10.1038/s42003-019-0389-3)
Supplement: Supplementary file 3 — Supplementary information [file 42003_2019_389_MOESM3_ESM.pdf]

Supplementary Figure 1

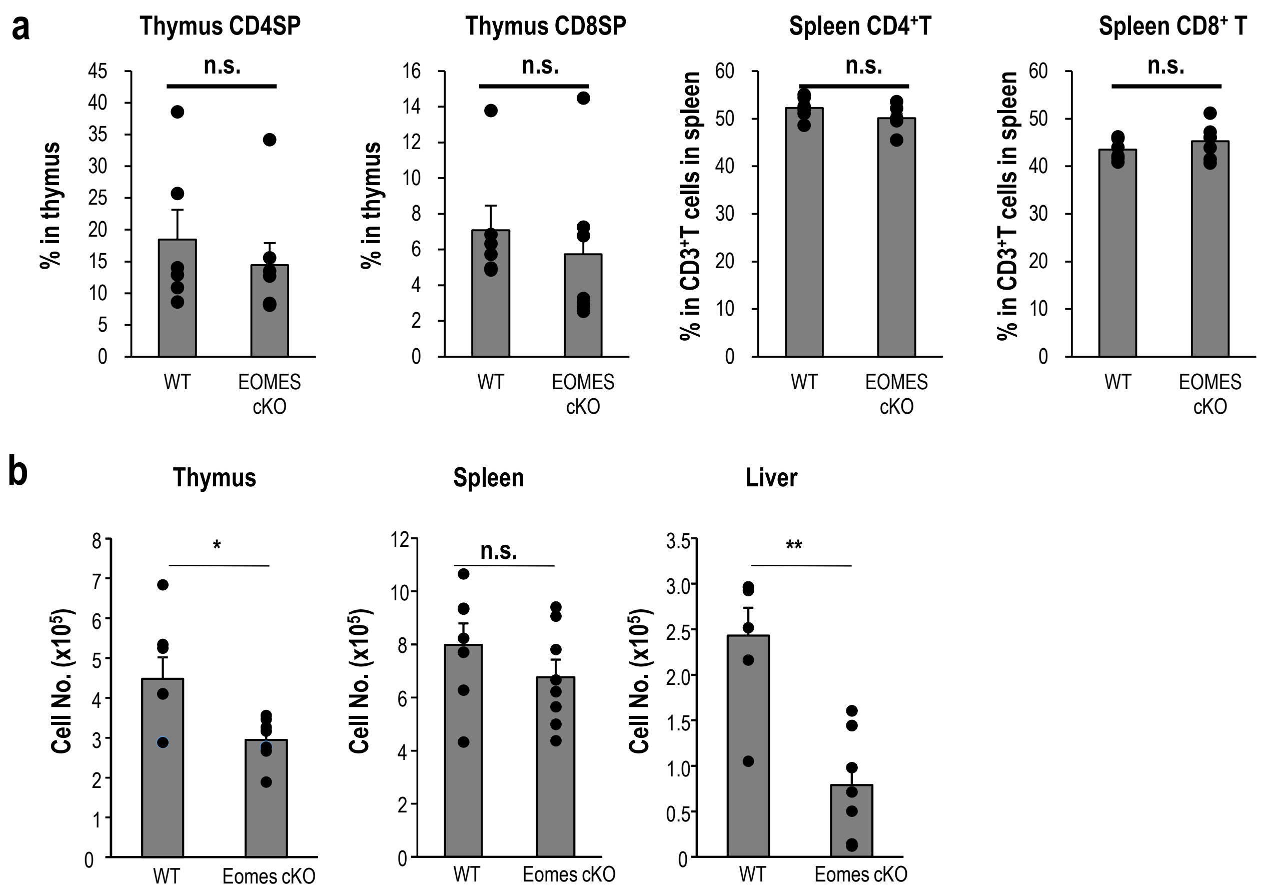

Supplementary Figure 1. The frequencies and cell numbers of T cells and iNKT cell in thymus, spleen and liver of WT and Eomes cKO

a Percentages of CD4 single positive (CD4SP) and CD8 single positive (CD8SP) cells in the thymus, splenic $CD4^+$ T and $CD8^+$ T cells of WT and Eomes cKO mice. (n = 6-8, mean ± SEM).

b Absolute cell number of iNKT cells in the thymus, spleen and liver of WT and Eomes cKO mice. (n = 6-8, mean ± SEM). Not significant; n.s., *p<0.05, **p<0.01, Mann-Whitney

Supplementary Figure 2

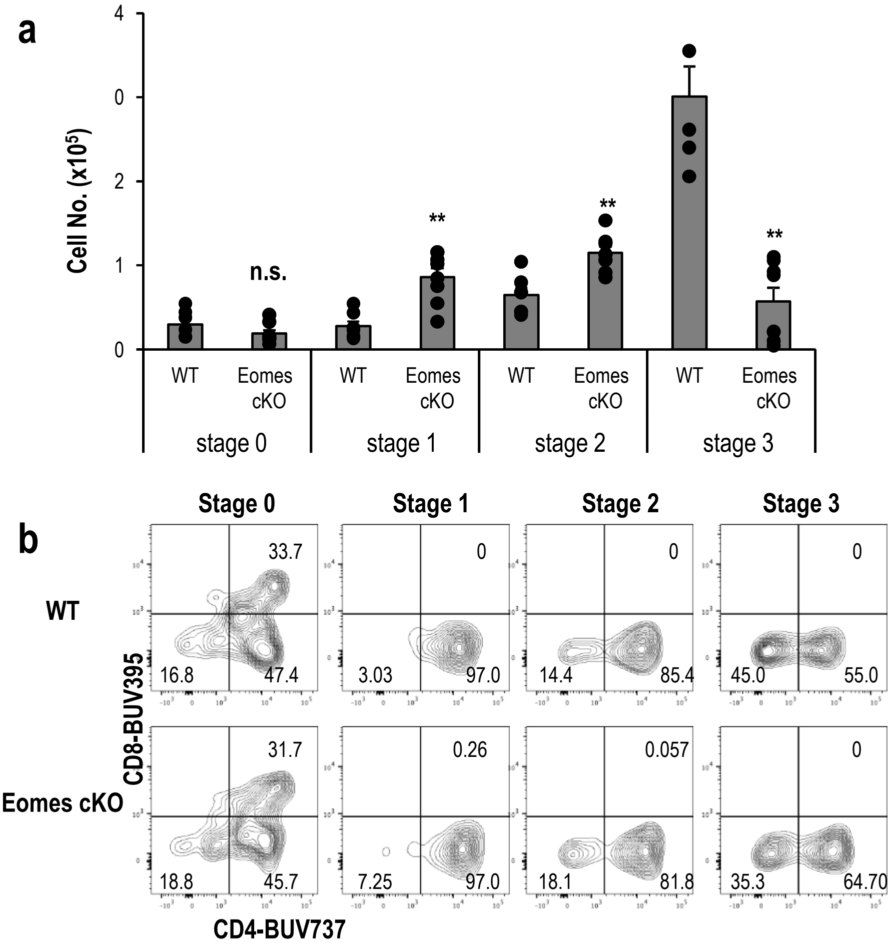

Supplementary Figure 2. Loss of Eomes results in a block in iNKT cell development
a Absolute cell number of iNKT cells at each stage in thymus of WT and Eomes cKO mice. (n = 6-8, mean ± SEM). Not significant; n.s., **p<0.01, Mann-Whitney
b. CD4 and CD8 expression by iNKT cells at each stage in thymus of WT and Eomes cKO mice. Data are representative of two experiments.

Supplementary Figure 3

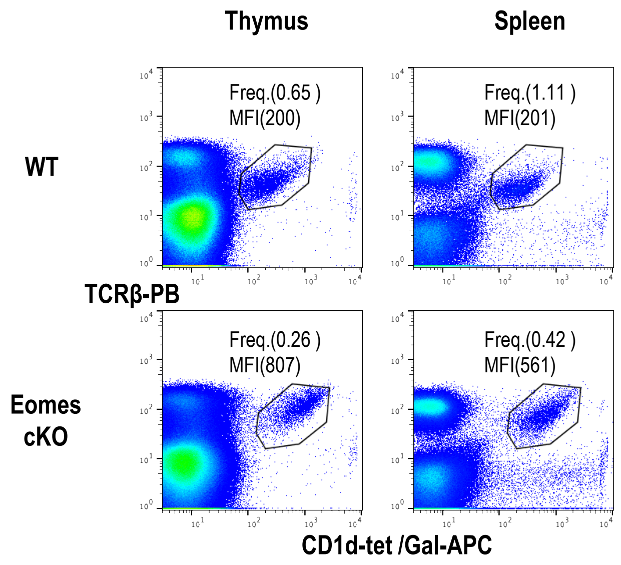

Supplementary Figure 3. Loss of Eomes results in altered Vα14TCR expression by iNKT cells

Expression of Vα14TCR in WT and Eomes cKO mice was assessed by frequency and mean fluorescence intensity (MFI) of FACS analyses. Data shown are representative from 3 experiments.

Supplementary Figure 4

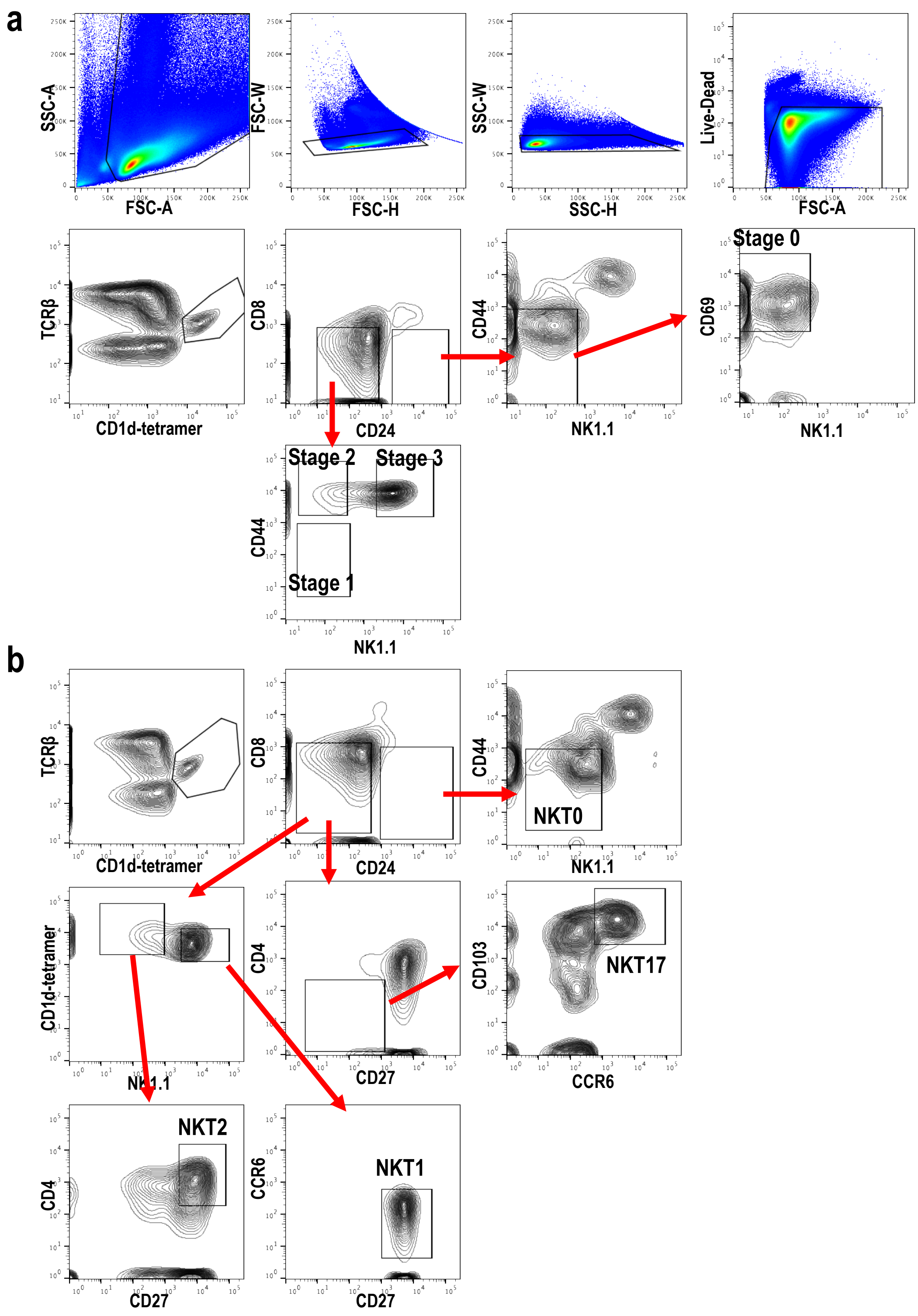

Supplementary Figure 4. Cell-sorting strategy

a. Sorting strategy for isolating Stage 0, 1, 2 and 3 iNKT cells from thymus.

b. Sorting strategy for isolating NKT0, 1, 2 and 17 subset from thymus.

Supplementary Table 1. The specific Primer sequences used for Chip-qPCR

Gene	Forward	Reverse
Hba-a1	acccaggggaagcacaac	tgcttgctctacccagtaaaactt
Gapdh	ggagctgagtcatggtggtt	tggaaggggctgctactaca
Eomes promoter	acaagtttccaagcggtcaa	aaccagccatttcctctcg
Eomes +1.6kb	tagcgaaagagggctagctg	gcttcttttcaaacacttgtgc
Eomes -0.6kb	gaagcccacttcagaagctaga	tccagagatggcaaaaggat
